# Direct Binding of Synaptopodin 2-Like Protein to Alpha-Actinin Contributes to Actin Bundle Formation in Cardiomyocytes

**DOI:** 10.3390/cells13161373

**Published:** 2024-08-17

**Authors:** Hiroshi Yamada, Hirona Osaka, Nanami Tatsumi, Miu Araki, Tadashi Abe, Keiko Kaihara, Ken Takahashi, Eizo Takashima, Takayuki Uchihashi, Keiji Naruse, Kohji Takei

**Affiliations:** 1 Department of Neuroscience, Graduate School of Medicine, Dentistry and Pharmaceutical Sciences, Okayama University, 2-5-1 Shikata-cho, Okayama 700-8558, Japantabe@md.okayama-u.ac.jp (T.A.); kohji@md.okayama-u.ac.jp (K.T.); 2 Graduate School of Science, Nagoya University, Furo-cho, Chikusa-ku, Nagoya 464-8601, Japanuchihashi.takayuki.z6@f.mail.nagoya-u.ac.jp (T.U.); 3 Department of Cardiovascular Physiology, Graduate School of Medicine, Dentistry and Pharmaceutical Sciences, Okayama University, 2-5-1 Shikata-cho, Okayama 700-8558, Japantakah-k2@okayama-u.ac.jp (K.T.); knaruse@md.okayama-u.ac.jp (K.N.); 4 Division of Malaria Research, Proteo-Science Center, Ehime University, 3 Bunkyo-cho, Matsuyama 790-8577, Japan; takashima.eizo.mz@ehime-u.ac.jp

**Keywords:** SYNPO2L, actinin, actin, sarcomere, cardiomyocyte

## Abstract

Synaptopodin 2-like protein (SYNPO2L) is localized in the sarcomere of cardiomyocytes and is involved in heart morphogenesis. However, the molecular function of SYNPO2L in the heart is not fully understood. We investigated the interaction of SYNPO2L with sarcomeric α-actinin and actin filaments in cultured mouse cardiomyocytes. Immunofluorescence studies showed that SYNPO2L colocalized with α-actinin and actin filaments at the Z-discs of the sarcomere. Recombinant SYNPO2La or SYNPO2Lb caused a bundling of the actin filaments in the absence of α-actinin and enhanced the α-actinin-dependent formation of actin bundles. In addition, high-speed atomic force microscopy revealed that SYNPO2La directly bound to α-actinin via its globular ends. The interaction between α-actinin and SYNPO2La fixed the movements of the two proteins on the actin filaments. These results strongly suggest that SYNPO2L cooperates with α-actinin during actin bundle formation to facilitate sarcomere formation and maintenance.

## 1. Introduction

The sarcomere is the morphological and functional contractile unit in striated skeletal and cardiac muscle and is located between two adjacent Z-discs in myofibrils. Cytoskeletal proteins such as actin and myosin help maintain the structure of the sarcomere. Alpha-actinin 2, a major component of Z-discs, forms antiparallel rod-shaped dimers that crosslink actin filaments and stabilize the contractile apparatus in the sarcomere [1].

The synaptopodin family of proteins contains at least three members, namely synaptopodin, synaptopodin 2, and synaptopodin 2-like protein (SYNPO2L). Synaptopodin 2 and SYNPO2L are highly expressed in skeletal and cardiac muscle. These proteins localize at the Z-discs of the sarcomere and are involved in the morphogenesis and function of skeletal and heart muscle [2]. SYNPO2L consists of two isoforms encoded by a single gene, SYNPO2La (long form, 978 amino acids) and SYNPO2Lb (short form, 749 amino acids) in mice [3]. Both proteins have a nuclear localization signal, and SYNPO2La has an N-terminal PDZ domain. SYNPO2La is predominantly expressed in adult skeletal and heart muscle. The expression of SYNPO2Lb is mainly restricted to the early developmental stage of skeletal and cardiac muscle. Knockdown of SYNPO2La in zebrafish results in decreased heart contractility and impaired formation of skeletal muscle [3]. Furthermore, overexpression of SYNPO2Lb in mice causes decreased muscle contractility [4], suggesting that SYNPO2L is crucial for the formation and maintenance of the sarcomere. SYNPO2L and other members of the synaptopodin family can bind to α-actinin [2,3]. However, the mechanism by which SYNPO2L interacts with actin in the Z-discs of the sarcomere remains to be clarified.

In the present study, we analyzed the interaction of SYNPO2L with actin filaments and α-actinin in vitro. We demonstrated that both SYNPO2La and SYNPO2Lb could directly bind to α-actinin, and the interaction between SYNPO2La and α-actinin fixed their movement on the actin filament. In addition, SYNPO2L in the absence of α-actinin caused actin filaments to form bundles and enhanced the formation of α-actinin-mediated actin bundles. These results suggest that SYNPO2L has an essential role in stabilizing the morphology of Z-discs by directly bundling actin filaments and enhancing the effect of α-actinin-mediated bundle formation.

## 2. Materials and Methods

### 2.1. Antibodies and Reagents

Rabbit anti-SYNPO2L antibody (cat# 21480-1-AP) was purchased from Proteintech Group Inc. (Rosemont, IL, USA). Mouse monoclonal anti-α-actinin (clone EA-53, cat# A7811) was purchased from Merck KgaA (Darmstadt, Germany). Alexa Fluor 488-conjugated donkey anti-rabbit IgG (cat# A21206), Alexa Fluor 555-conjugated donkey anti-rabbit IgG (cat# A31572), Alexa Fluor 555-conjugated donkey anti-mouse IgG (cat# A31570), Alexa Fluor 568-conjugated donkey anti-goat IgG (cat# A11057), Alexa Fluor 488-conjugated goat anti-mouse IgG antibody (cat# A11001), Alexa Fluor 594-conjugated goat anti-rabbit IgG antibody (cat# A11037) and Alexa Fluor 488-labeled phalloidin (cat# A12379) were obtained from Thermo Fisher Scientific (Waltham, MA, USA). 

### 2.2. Isolation and Cell Culture of Mouse Cardiomyocytes

First, the plates were prepared for cell seeding. Specifically, one sterile glass coverslip measuring 1 cm × 1 cm was placed into each well of a 24-well plate. Subsequently, 0.5 mL of a 50-fold diluted collagen solution (cat# IPC-30, KOKEN, Tokyo, Japan) was added to each well. The plates were then incubated at 37 °C for 1 h, followed by several washes of the glass coverslips with distilled water and then air-drying. All animal handling and experimental procedures were approved by the Animal Care and Use Committee of Okayama University. Primary cardiomyocytes were isolated from the ventricles of neonatal mice (C57BL/6J, CLEA Japan, Shizuoka, Japan) on postnatal day 1. The mice were euthanized by decapitation under isoflurane anesthesia, then the hearts were removed, the ventricles were dissected, and the atria were removed. The ventricular tissue was subsequently cut into several fragments of approximately 2 mm^2^ in size. These ventricular tissue fragments were gently agitated in 10 mL of phosphate-buffered saline and washed three times. The tissue was then subjected to enzymatic digestion using 0.06% trypsin (10 mL) at 37 °C for 10 min with gentle agitation, and the enzyme treatment was repeated three times. The supernatant containing the suspended cells was promptly supplemented with DMEM containing 10% FCS to halt trypsinization, and the cells were collected by centrifugation at 400× *g* for 5 min. The cells were re-suspended in fresh DMEM containing 10% FCS and preincubated at 37 °C for 45 min to remove the fibroblasts. Subsequently, the cells were seeded into collagen-coated 24-well plates (5 × 10^4^ cells per well) prepared as described above and cultured at 37 °C under 5%CO_2_.

### 2.3. Purification of Recombinant Proteins

His-tagged human SYNPO2La or SYNPO2Lb were expressed using a wheat germ cell-free expression system (CellFree Sciences, Matsuyama, Japan), purified by immobilized-nickel affinity chromatography with Ni Sepharose™ 6 Fast Flow (Cat#17531802, Cytiva, Marlborough, MA, USA). These proteins were resolved in 100 mM NaCl, 50 mM Tris-HCl, 500 mM imidazole, pH 8.0, and stored at 4 °C until use.

### 2.4. Fluorescent Microscopy

Cultured cardiomyocytes (5 × 10^4^ cells/coverslip) were fixed with 4% paraformaldehyde in phosphate-buffered saline containing 0.2 mM CaCl_2_ and 2 mM MgCl_2_ (PBS (+)) for 20 min, then permeabilized with 0.1 % Triton-X100 for 15 min. Samples were incubated in 10% donkey serum in PBS (+) for 1 h and were then treated with anti-SYNPO2L (1:50) and/or anti-α-actinin (1:250) for 1 h. After washing with PBS (+) five times every 5 min, the samples were further treated with secondary antibodies for 1 h. After washing, the samples were mounted in PermaFluor (Thermo Fisher Scientific, Waltham, MA, USA) [5]. SYNPO2L, α-actinin, and actin filaments were visualized by double immunofluorescence. Samples were examined using a spinning-disc confocal microscope system (X-Light Confocal Imager; CREST OPTICS S.P.A., Rome, Italy) combined with an inverted microscope (IX-71; Olympus Optical Co., Ltd., Tokyo, Japan) and an iXon+ camera (Oxford Instruments, Oxfordshire, UK). The confocal system was controlled by MetaMorph software, version 7.10.3.279 (Molecular Devices, San Jose, CA, USA). When necessary, the images were processed using Adobe Photoshop 2024 or Adobe Illustrator 2024 software.

### 2.5. Electron Microscopy

For negative staining of the indicated proteins, reaction mixtures containing 1 μM α-actinin, SYNPO2La or SYNPO2Lb, and 4 μM actin filaments were incubated at room temperature for 3 h. Rabbit skeletal muscle-derived α-actinin (cat# AT01-A, Cytoskeleton Inc., Denver, CO, USA) was reconstituted in F buffer (100 mM KCl, 10 mM Tris-HCl, 0.2 mM CaCl_2_, 1 mM MgCl_2_, 1 mM DTT, 0.2 mM ATP, pH 7.4). Actin (APHL99, Cytoskeleton Inc.) was polymerized in F buffer for 1 h. The samples were absorbed into a Formvar- and carbon-coated copper grid and then stained with 3% uranyl acetate in ddH_2_O for 2 min. Electron microscopy was carried out using a Hitachi H-7650 transmission electron microscope (TEM) (Hitachi High-Tech Science Corporation, Tokyo, Japan).

### 2.6. Morphometry

To quantify the length of the α-actinin dimer, the sample was negatively stained and observed by TEM. TEM images were taken at high magnification (×20,000). The 10 randomly selected α-actinin dimer in the TEM image (512 × 512 pixels, 8 images) was quantified using Image J software version 1.52a.

To quantify actin bundle formation, the reaction mixture was negatively stained and observed by TEM. TEM images were taken at low magnification (×300). The area corresponding to the actin bundles in the TEM images (512 × 512 pixels) was quantified using Image J software version 1.52a. The quantification was based on either 48 images (α-actinin/F-actin), 19 images (SYNPO2La/F-actin), 29 images (SYNPO2La/α-actinin/F-actin), 20 images (SYNPO2Lb/F-actin), or 20 images (SYNPO2Lb/α-actinin/F-actin) from three or more independent experiments.

### 2.7. High-Speed Atomic Force Microscopy (AFM)

High-speed AFM was conducted using a custom-built, tapping mode-based instrument. An Olympus microcantilever (cat# AC10, Tokyo, Japan) with a resonance frequency of approximately 500 kHz and a spring constant of approximately 0.1 N/m in liquid was used. Since the AC10 microcantilever does not have a sharpened tip, we used an amorphous carbon tip deposited by electron-beam deposition on the cantilever end. The equipment is described in detail in a previous paper [6].

The proteins α-actinin and SYNPO2La were observed using bare mica substrates; α-actinin solution or SYNPO2La was placed on a freshly cleaved mica surface, incubated for 3 min, and then washed with observation buffer (5 mM Tris-HCl (pH 8.0), 0.2 mM CaCl_2_, 0.5 mM DTT, 100 mM KCl, 2 mM MgCl_2_) to remove excess molecules immediately before high-speed AFM observations. To observe the interaction between α-actinin and SYNPO2La, α-actinin was first adsorbed onto the mica substrate, then SYNPO2La was added to the observation buffer during high-speed AFM, and the binding of SYNPO2La to α-actinin was observed after a few min. The stock concentrations of α-actinin and SYNPO2La were 10 μM and 2.8 μM, respectively, and these were appropriately diluted for the measurements. The specific concentrations used during the observation are provided in the figure captions.

A positively charged lipid bilayer was used as a substrate to retain actin filaments. This was to hold the negatively charged actin filaments to the substrate while preventing strong adsorption of α-actinin and SYNPO2La to the substrate. Neutral phospholipid DPPC and positively charged phospholipid DATAP were dissolved in chloroform and mixed at a molar ratio of DPPC:DPTAP = 7:3, after which the chloroform was evaporated. Ultra-pure water was added to adjust the concentration to 1 mg/mL, and the lipid vesicle suspension was diluted to 0.1 mg/mL with 5 mM MgCl_2_ solution and subjected to about 20 rounds of sonication using a tip sonicator. Then, 2 μL of the prepared lipid solution was dropped onto the mica surface, incubated for 10 min to develop the lipid bilayer, and then washed with 80 μL of observation buffer to remove the excess vesicles. Next, 2 μL of 7 μM actin filaments were dropped onto the lipid bilayer substrate, incubated for 5 min, and the substrate surface was washed again with 80 μL of observation buffer. When adding α-actinin or SYNPO2La, the injection was performed into the observation buffer within the cantilever holder. All observations were conducted at room temperature.

### 2.8. Ethics and Animal Use Statement

All experiments and protocols were approved by the institutional Animal Care and Use Committee of Okayama University (OKU-2024424). All efforts were made to minimize animal suffering. Mice were euthanized under anesthesia before the whole hearts were removed.

### 2.9. Statistical Analysis

Data were analyzed for statistical significance using KaleidaGraph software for Macintosh, version 4.1 (Synergy Software Inc., Reading, PA, USA). An analysis of variance and Tukey’s honest significant difference post hoc test was used to compare several groups. *p* values of less than 0.05 (*) or 0.01 (**) were considered significant. 

## 3. Results

### 3.1. SYNPO2L Colocalizes with α-Actinin and Actin Filaments at Sarcomeric Z-Discs

**Figure 1:**
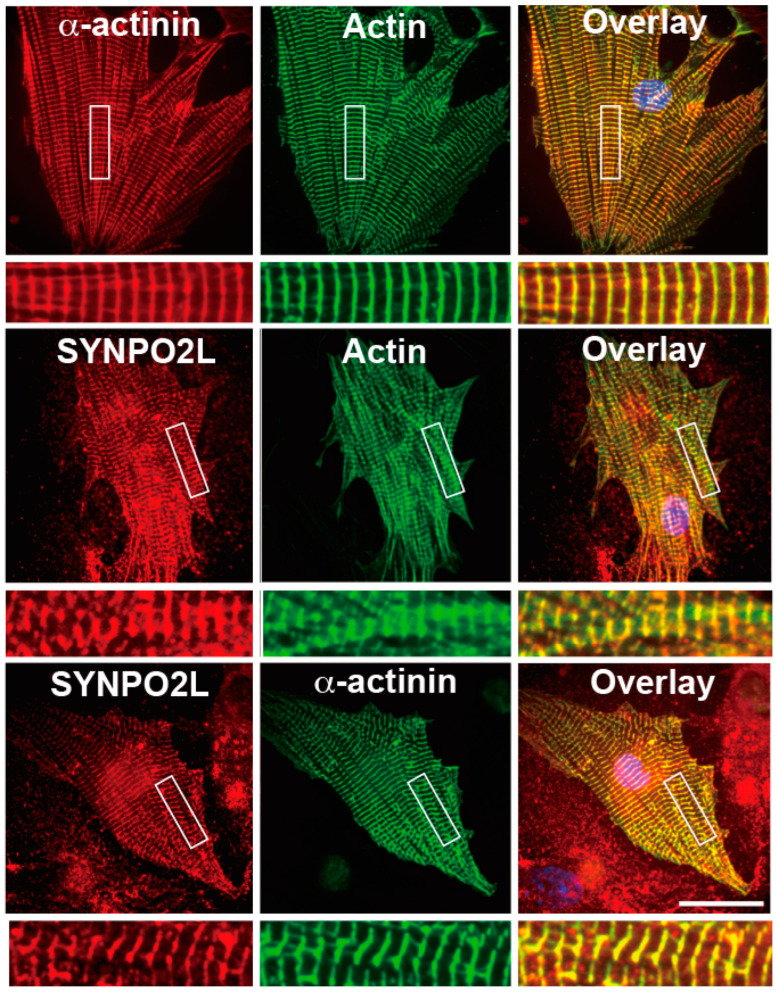
SYNPO2L, α-actinin, and actin filaments accumulate at the Z-discs of the sarcomere in mouse cardiomyocytes. Double immunofluorescence images of α-actinin and actin filaments (**upper** panels), SYNPO2L and actin filaments (**middle** panels) or SYNPO2L and α-actinin (**bottom** panels) in mouse cardiomyocytes. Areas indicated by rectangles are enlarged in the underneath image. Bar: 30 μm.

We first investigated the localization of SYNPO2L in cultured mouse cardiomyocytes using double immunofluorescence experiments. SYNPO2L was colocalized with actin filaments and α-actinin, a marker of sarcomeric Z-discs (Figure 1). 

### 3.2. SYNPO2La or SYNPO2Lb Alters the Shape of the α-Actinin Dimer and Its Ability to Facilitate the Formation of Actin Bundles

**Figure 2:**
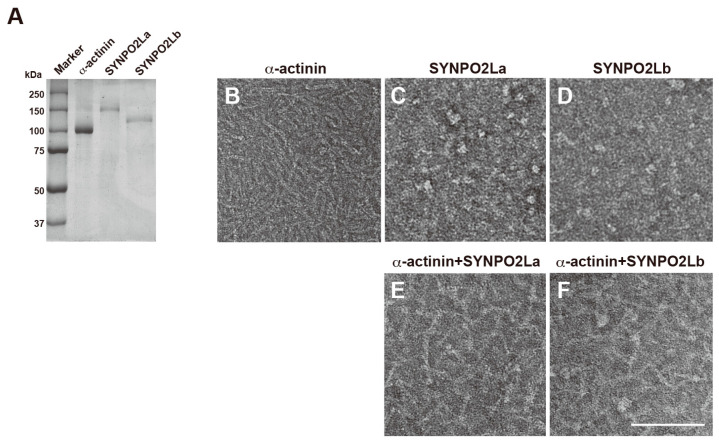
SYNPO2La or SYNPO2Lb alters the shape of the α-actinin dimer in vitro. (**A**) SDS-PAGE of α-actinin, SYNPO2La or SYNPO2Lb. Alpha-actinin (5 μg), SYNPO2La (5 μg), and SYNPO2Lb (10 μg) were analyzed and visualized by SYPRO Orange staining. (**B**–**F**) Morphological changes of α-actinin dimers in the presence of SYNPO2La or SYNPO2Lb. One micromolar of α-actinin (**B**), SYNPO2La (**C**), SYNPO2Lb (**D**), α-actinin and SYNPO2La (**E**) or α-actinin and SYNPO2Lb (**F**) was incubated for 3 h at room temperature. The samples negatively stained and observed by TEM. Scale bar: 100 nm.

The fact that SYNPO2L colocalizes with both α-actinin and actin filaments in mouse cardiomyocytes indicates that the three proteins might interact with one another and be involved in actin remodeling to maintain the structure of the sarcomere. To test this possibility comprehensively, we prepared recombinant SYNPO2La and SYNPO2Lb, and examined their effect on α-actinin and actin filaments. SYPRO Orange staining revealed that α-actinin, recombinant SYNPO2La, and recombinant SYNPO2Lb were highly purified (Figure 2A). TEM of negatively stained samples showed that α-actinin in the absence of other proteins was cylindrically shaped and 35.6 ± 0.83 nm in length (Figure 2B); this finding is consistent with previous reports showing that α-actinin dimers have an elongated straight shape [1,7]. On the other hand, SYNPO2La alone and SYNPO2Lb alone did not have cylindrical or linear structures; instead, these proteins had a globule form, and the SYNPO2La and SYNPO2Lb globules were of various sizes (Figure 2C,D). When α-actinin was mixed with SYNPO2La or SYNPO2Lb, the protein complexes were cylindrically shaped, and the majority of the structures were visibly different from the structure of α-actinin alone (Figure 2E,F). These results strongly suggest that SYNPO2La and SYNPO2Lb directly interact with α-actinin to form complexes.

**Figure 3:**
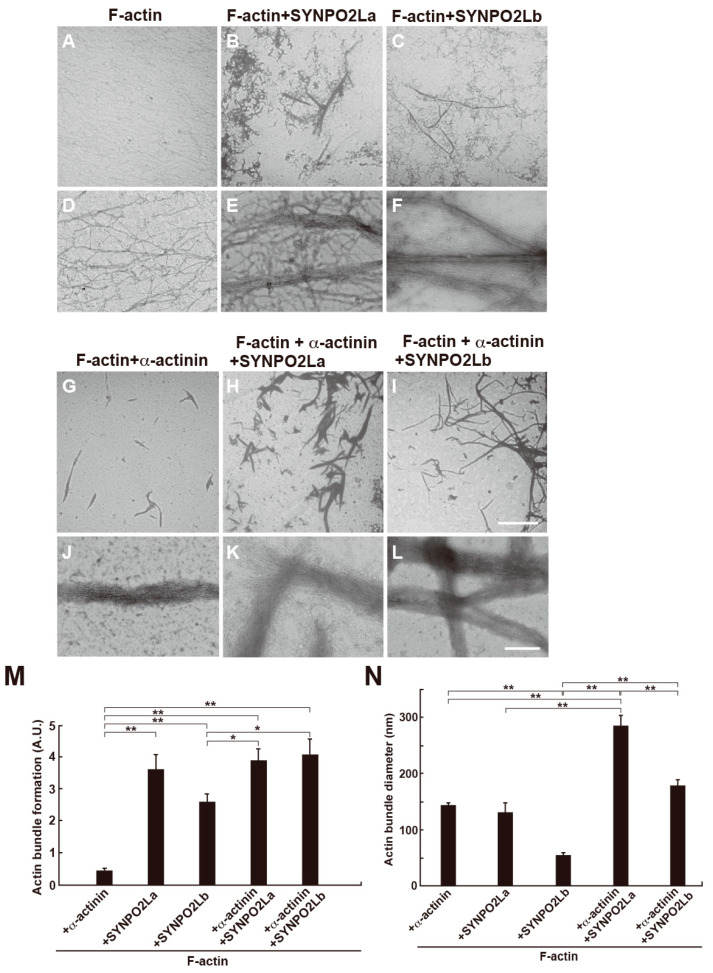
SYNPO2La or SYNPO2Lb directly bundles actin filaments and SYNPO2La causes the formation of thick actin bundles. One micromolar α-actinin, SYNPO2La, and SYNPO2Lb were mixed with 4 μM preformed actin filaments and incubated for 3 h at room temperature. The mixture was negatively stained and assessed by TEM at low magnification (**A**–**C**,**G**–**I**) or high magnification (**D**–**F**,**J**–**L**). Actin filaments alone (**A**,**D**). The presence of SYNPO2La or SYNPO2Lb enhanced the formation of thick and long actin bundles. Scale bar: 5 μm in (**A**–**C**,**G**–**I**), 300 nm in (**D**–**F**,**J**–**L**). (**M**) Morphometric analysis of actin bundle formation by the indicated proteins. The formation of actin filament bundles was determined by densitometry using Image J analysis of TEM images taken at low magnification (×300). The results were obtained from three independent experiments. * *p* < 0.05, ** *p* < 0.01. (**N**) The diameter of actin bundles formed by the indicated proteins. The diameter of each actin bundle was measured at three different sites of the TEM image taken at high magnification (×20,000). The results were obtained from three independent experiments. ** *p* < 0.01.

Next, we investigated the interaction of SYNPO2La or SYNPO2Lb with the actin filaments. The actin filaments alone were uniformly dispersed and did not form bundles (Figure 3A,D). As reported previously, α-actinin facilitated the formation of actin bundles [8] (Figure 3G,J). The presence of SYNPO2La or SYNPO2Lb also caused the formation of actin bundles that had diameters of 131.8 ± 15.9 nm or 54.8 ± 4.0 nm, respectively (Figure 3B,C,E,F,M,N), suggesting that both SYNPO2La and SYNPO2Lb could directly bundle the actin filaments. Furthermore, the actin bundles that formed when α-actinin and actin/SYNPO2La were combined had larger diameters than those formed when α-actinin and actin were combined (Figure 3H,K,M,N). The diameter of actin bundles formed when α-actinin and actin/SYNPO2Lb were combined were of a similar size to those formed in the presence of α-actinin and actin (Figure 3I,L,M,N).

### 3.3. High-Speed AFM Shows That SYNPO2La Interacts with α-Actinin, and the Complex Contributes to Actin Bundle Formation

**Figure 4:**
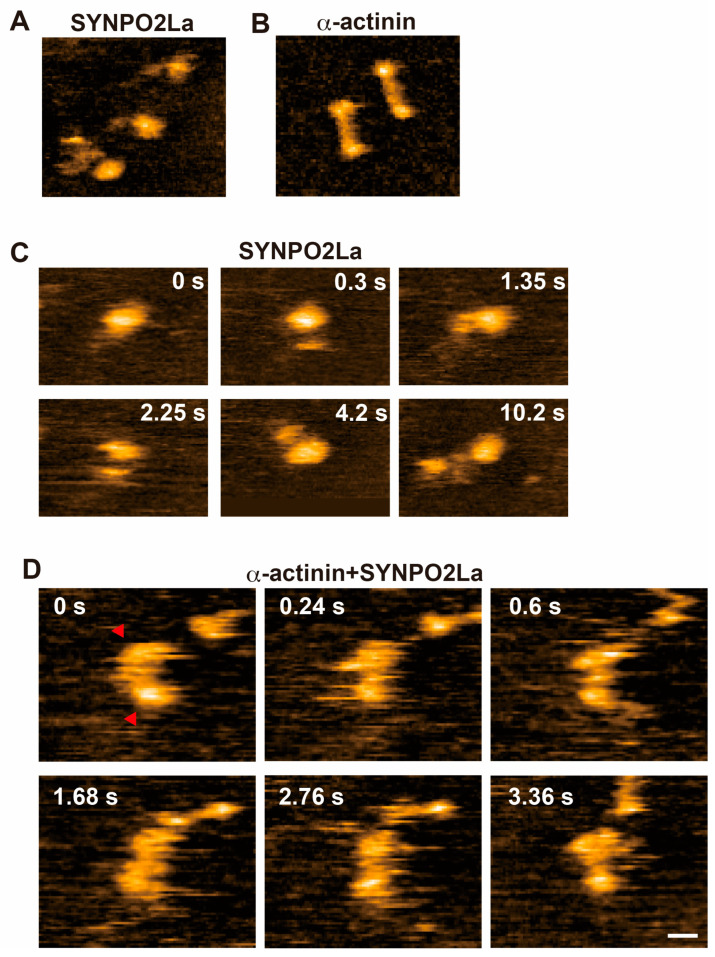
Spatio-temporal high-speed AFM observations of SYNPO2La binding to α-actinin. (**A**) SYNPO2La (11 nM) formed various shapes and sometimes had a globular shape. (**B**) Alpha-actinin (50 nM) formed dimers that had a globular shape at both ends. (**C**) Time-lapsed high-speed AFM images of SYNPO2La (11 nM). (**D**) Time-lapsed high-speed AFM images of α-actinin (50 nM)/SYNPO2La (28 nM). Scale bar in (**A**–**D**): 20 nm.

**Figure 5:**
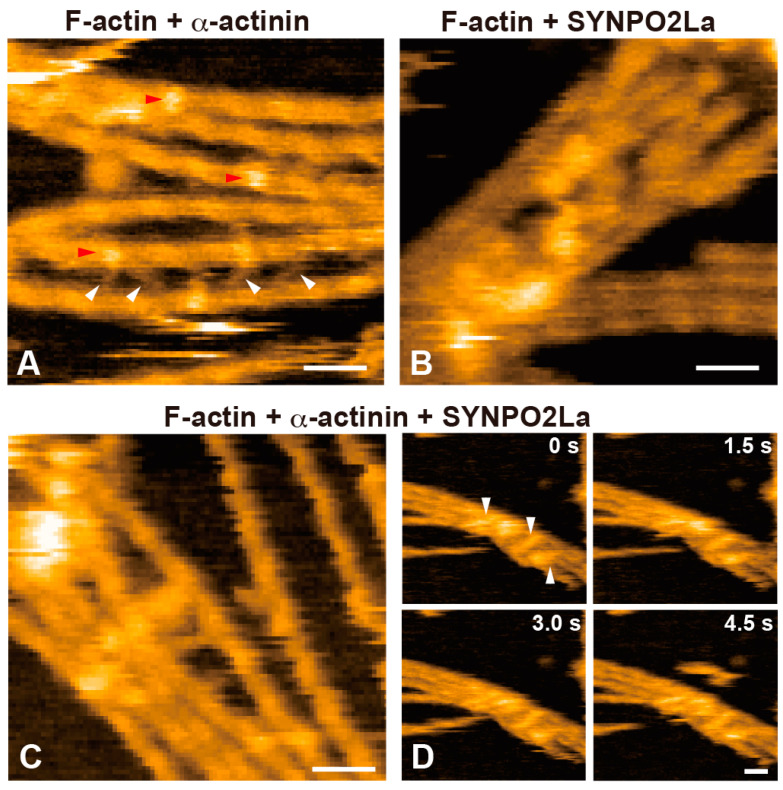
Spatio-temporal characteristics of actin-filament crosslinking mediated by α-actinin and/or SYNPO2La. (**A**) High-speed AFM image of α-actinin (140 nM)/actin filaments. Alpha-actinin caused the crosslinking of adjacent actin filaments into a ladder-like structure (indicated by the white arrowheads) and bound to the actin filament (indicated by the red arrowheads). Scale bar: 50 nm. (**B**) High-speed AFM image of SYNPO2La (120 nM)/actin filaments. SYNPO2La forms aggregates, which are bound to and crosslinked actin filaments. Scale bar: 50 nm. (**C**) High-speed AFM image of α-actinin (140 nM)/SYNPO2La (200 nM)/actin filaments. Globular, rod-like or complex shapes were observed on the actin filaments. Scale bar: 50 nm. (**D**) Time-lapsed high-speed AFM images of α-actinin/SYNPO2La/actin filaments. The α-actinin/SYNPO2La complex that was bound to actin filaments rarely moved along bundled actin filaments (indicated by the white arrowheads). Scale bar: 50 nm.

Since SYNPO2La greatly enhanced α-actinin-mediated actin bundle formation (Figure 3), we investigated the spatio-temporal dynamics of SYNPO2La activity using high-speed AFM. Consistent with the observation from negatively stained TEM (Figure 2C), SYNPO2La alone appeared as a globule structure with an amorphous tail (Figure 4A,C). This amorphous tail was highly flexible, and interconvertible with the globular structure, as seen in successive images (Figure 4C and Supplementary Movie S1). By contrast, α-actinin alone was stable and rod-shaped with bulbous ends (Figure 4B); these properties are comparable with the reported structure of the α-actinin homodimer [1]. When SYNPO2La and α-actinin coexisted on the mica substrate, the rod-shaped structure became mobile, and both ends of the rod were often decorated with globules (Figure 4D and Supplementary Movie S2). The globules had similar properties to those formed by SYNPO2La alone (Figure 4A), and the fusion of the rod and the globule was occasionally observed (Supplementary Movie S3). Next, the effects of the interaction between SYNPO2La and α-actinin on actin bundling were determined. SYNPO2La was mixed with preformed actin filaments that were incubated with α-actinin. As shown in Figure 5A, α-actinin crosslinked the adjacent actin filaments, and α-actinin dynamically repeated dissociation and binding to actin filaments (Figure 5A and Supplementary Movie S4). SYNPO2La formed clusters, which bound to several actin filaments and caused the bundling of these filaments (Figure 5B). We next studied the combination of α-actinin/SYNPO2La and actin filaments, in which the actin filaments were crosslinked by α-actinin, and we observed many bright spots on the actin filaments that corresponded to SYNPO2La or α-actinin/SYNPO2La (Figure 5C). The α-actinin/SYNPO2La complex on the bundled actin filaments rarely moved (Figure 5D and Supplementary Movie S5). These results suggest that the α-actinin/SYNPO2La complex causes the bundling of actin filaments, and the interaction between SYNPO2La and α-actinin helps to stabilize α-actinin on actin filaments.

## 4. Discussion

SYNPO2La and SYNPO2Lb are expressed and colocalized with α-actinin and actin filaments at sarcomeric Z-discs in mouse cardiomyocytes [3]. In the current study, we investigated the role of SYNPO2L by determining the direct interactions between SYNPO2L, α-actinin, and actin filaments in vitro. Double immunofluorescence studies confirmed that these three proteins are colocalized at sarcomeric Z-discs in mouse cardiomyocytes (Figure 1). Recombinant SYNPO2La or SYNPO2Lb formed complexes with α-actinin (Figure 2). Furthermore, SYNPO2La or SYNPO2Lb caused direct bundling of actin filaments. In particular, SYNPO2La enhanced α-actinin-mediated formation of actin bundles (Figure 3). Finally, high-speed AFM observations showed that SYNPO2La directly bound to α-actinin (Figure 4), and the α-actinin, SYNPO2La, and SYNPO2La/α-actinin complex crosslinked the adjacent actin filaments. Furthermore, the interaction between SYNPO2La and α-actinin fixed their movements on the actin filament (Figure 5).

### 4.1. SYNPO2L Directly Binds to and Bundles Actin Filaments

SYNPO2L is expressed in cardiac muscle, skeletal muscle, and smooth muscle [3]. In cardiomyocytes, SYNPO2L is localized at sarcomeric Z-discs, in which actin and actin-related proteins such as α-actinin and titin are enriched. These proteins likely regulate the actin cytoskeleton to maintain the structure of Z-discs [9]. Beqqali and colleagues used immunoprecipitation to show that SYNPO2Lb interacts with α-actinin in COS-1 cells that overexpress SYNPO2Lb or in C2C12 myotubes [3]. In the current study, we demonstrated that SYNPO2La or SYNPO2Lb bound to actin filaments in the absence of α-actinin, which resulted in the formation of actin bundles in vitro (Figure 3 and Figure 5). In addition, SYNPO2L bundles actin filaments via interaction between SYNPO2L and α-actinin. It seems that SYNPO2L isoforms act not only in the absence of other actin-binding proteins but also in collaboration with α-actinin on actin, similar to other synaptopodin family proteins [10,11,12]. As both SYNPO2La and SYNPO2Lb could bundle actin (Figure 3), SYNPO2L may have several actin-binding sites and/or an increase in the number of actin-binding sites caused by self-polymerization. Although both SYNPO2La and SYNPO2Lb interacted with α-actinin (Figure 2), SYNPO2Lb did not enhance α-actinin-mediated actin bundle formation, in contrast to the additive effect observed with SYNPO2La (Figure 3). SYNPO2Lb is expressed in the embryonic heart, while SYNPO2La is expressed mainly in the adult heart [3]. Furthermore, the overexpression of SYNPO2Lb in mice caused heart dysfunction and perturbed Z-disc morphology [4]. Taken together, these findings suggest that the different effects of SYNPO2La and SYNPO2Lb on α-actinin-dependent formation of actin bundles may reflect the existence of isoform-specific functions, although further work is needed to clarify this hypothesis.

### 4.2. SYNPO2La May Bind to the Globular Domain of Antiparallel α-Actinin Dimers and Help Stabilize α-Actinin

In the present study, the binding of the globular form of SYNPO2La to α-actinin was captured by high-speed AFM (Figure 4). Alpha-actinin formed antiparallel dimers that had a globular shape at both ends, and these globular regions crosslinked the actin filaments [1]. We showed that SYNPO2La bound to the globular region of α-actinin dimers (Figure 4D and Supplementary Movie S3). In addition, the interaction between SYNPO2La and α-actinin fixed the movements of the two proteins on the actin filaments (Figure 5D). Therefore, it is conceivable that the interaction of SYNPO2La with α-actinin might affect the crosslinking ability of α-actinin. As the α-actinin/SYNPO2La complex facilitated the formation of actin bundles, the interaction between α-actinin and SYNPO2La may contribute to the stability of α-actinin on actin filaments.

In the past several years, multiple genome-wide association studies have identified several genomic loci associated with atrial fibrillation, particularly that the loss-of-function of several actin-related proteins localized at Z-discs causes cardiac dysfunction [13]. A recent report indicates that loss-of-function variants in the SYNPO2L gene increase the risk of atrial fibrillation. However, the precise role of SYNPO2L at Z-discs in that study was not determined [14]. The effect of SYNPO2L on actin and α-actinin activity demonstrated in this study might represent an essential physiological function of SYNPO2L, and help elucidate the pathogenesis of atrial fibrillation, especially those forms caused by SYNPO2L variants.

## 5. Conclusions

This study shows that sarcomeric SYNPO2L bundles actin filaments in the absence of other actin-bundling proteins. In addition, the interaction between SYNPO2L and α-actinin enhances α-actinin-mediated actin bundling. In conclusion, SYNPO2L, α-actinin, and the SYNPO2L/α-actinin complex may work together in actin bundling for the maintenance of Z discs at the sarcomere.

## Data Availability

The raw data supporting the conclusions of this article will be made available by the authors, without undue reservation.

## Supplementary Materials

The following supporting information can be downloaded at: https://www.mdpi.com/article/10.3390/cells13161373/s1, Movie S1: Live imaging of SYNPO2La by high-speed AFM, Movie S2: Live imaging of SYNPO2La/α-actinin by high-speed AFM, Movie S3: Live imaging of fusion process of α-actinin to SYNPO2La by high-speed AFM, Movie S4: Live imaging of α-actinin/F-actin by high-speed AFM, Movie S5: Live imaging of α-actinin/SYNPO2La/F-actin by high-speed AFM.
